# The Effect of LED Light Spectra on the Growth, Yield and Nutritional Value of Red and Green Lettuce (*Lactuca sativa*)

**DOI:** 10.3390/plants12030463

**Published:** 2023-01-19

**Authors:** Abdullah A. Alrajhi, Abdulaziz S. Alsahli, Ibrahim M. Alhelal, Hail Z. Rihan, Michael P. Fuller, Abdullah A. Alsadon, Abdullah A. Ibrahim

**Affiliations:** 1 National Center for Agriculture Technology, King Abdulaziz City for Science and Technology, P.O. Box 6086, Riyadh 11442, Saudi Arabia; 2 Department of Agricultural engineering, College of Food and Agriculture Sciences, King Saud University, P.O. Box 2460, Riyadh 11451, Saudi Arabia; 3 School of Biological and Marine Sciences, Faculty of Science and Engineering, University of Plymouth, Plymouth PL4 8AA, UK; 4 Phytome Life Sciences, Launceston PL15 7AB, UK; 5 Department of Plant Production, College of Food and Agriculture Sciences, King Saud University, P.O. Box 2460, Riyadh 11451, Saudi Arabia

**Keywords:** controlled environment agriculture (CEA), light emitting diodes (LED), light spectra, lettuce, yield, photosynthetic rate, vitamin C

## Abstract

Controlled Environment Agriculture (CEA) is a method of increasing crop productivity per unit area of cultivated land by extending crop production into the vertical dimension and enabling year-round production. Light emitting diodes (LED) are frequently used as the source of light energy in CEA systems and light is commonly the limiting factor for production under CEA conditions. In the current study, the impact of different spectra was compared with the use of white LED light. The various spectra were white; white supplemented with ultraviolet b for a week before harvest; three combinations of red/blue lights (red 660 nm with blue 450 nm at 1:1 ratio; red 660 nm with blue 435 nm 1:1 ratio; red 660 nm with blue at mix of 450 nm and 435 nm 1:1 ratio); and red/blue supplemented with green and far red (B/R/G/FR, ratio: 1:1:0.07:0.64). The growth, yield, physiological and chemical profiles of two varieties of lettuce, Carmoli (red) and Locarno (green), responded differently to the various light treatments. However, white (control) appeared to perform the best overall. The B/R/G/FR promoted the growth and yield parameters in both varieties of lettuce but also increased the level of stem elongation (bolting), which impacted the quality of grown plants. There was no clear relationship between the various physiological parameters measured and final marketable yield in either variety. Various chemical traits, including vitamin C content, total phenol content, soluble sugar and total soluble solid contents responded differently to the light treatments, where each targeted chemical was promoted by a specific light spectrum. This highlights the importance of designing the light spectra in accordance with the intended outcomes. The current study has value in the field of commercial vertical farming of lettuce under CEA conditions.

## 1. Introduction

Environmental conditions present a major limiting factor for food production in most regions of the world and this is particularly acute in desert countries such as Saudi Arabia and most of the Middle East. High temperatures and water availability are major limiting factors in these regions, and intense irradiation often exceeds crop absorption capacity. As a consequence, “Controlled Environment Agricultural” (CEA) systems, or what is commonly called “Plant Factory” (PF) systems, could provide promising solutions for food security in these regions. Protected agriculture has developed from simple polytunnel structures to high-tech glasshouses and is now developing to completely CEA that optimizes the productivity and quality of plants. Major technical developments influenced by advances in precision technology, lighting, automation and data processing are making CEA systems a cost-efficient reality [1]. CEA systems offer greater predictability and increases in crop yield per unit area together with improvements in crop quality and improved shelf-life.

A PF is a typical vertical farming model based on a high-rise multi-level factory design where recycled water is supplemented with nutrients and used to feed plants through an advanced hydroponic system [2]. Light emitting diodes (LED) lighting systems are used as the sole source of light (energy) in the PF setting and the energy consumption of the PF can be offset using sustainable power generating systems such as photo-voltaics or wind turbines. Such PFs are being created as both research and production facilities.

Light has a great impact on the growth, yield, development, morphology and chemical composition of plants [3]. Light intensity (radiant density), light spectrum and photo-period all have significant impacts on plant growth and physiology [4]. LED lighting systems have unique features that enable them to be optimized for PF settings, including high light-use efficiency, long life span and high efficacy, leading to reduced associated heating [5] compared to other artificial lighting sources, such as fluorescent tubes or sodium vapor lamps. Furthermore, spectral specificity can be introduced through the design of the LED array by employing a mixture of LEDs with different wavelength emittance, controlled through appropriate control systems [6,7,8]. Such systems have a high degree of commercial applicability.

Plant species react differently to various wavelengths due to differences in their photoreceptors [9,10]. A significant amount of research has investigated the impact of LEDs on the growth, shape, yield and edible quality parameters of several plant species [6,7,11,12,13,14]. The impact of LEDs on the chemical composition, such as vitamin C content, chlorophyll content, soluble sugar, protein level and antioxidant activity has also been the subject of an increasing amount of recent research [15,16,17].

Both red (R) and blue (B) wavelengths are reported to have significant impacts on plant growth and development. These wavelengths are mainly absorbed by photosynthetic pigments, leading to carbon assimilation. However, these wavelengths can also have major effects on plant architecture and development [4]. Red induces transformations in phytochrome and is important for phytochemical synthesis, such as phenolics and oxalate. Red wavelengths are also essential for the development of the photosynthetic apparatus [18,19]. Blue is crucial for the development of chloroplasts and for photo-morphogenesis. Blue wavelengths are important in influencing stomatal opening and for the upregulation of chlorophyll and anthocyanin [20,21]. Green wavelengths, although not readily absorbed by the red/blue photosynthetic pigments, have an impact on the growth and development of several plants and it has been reported that the addition of green light to the red/blue LED arrays has a positive and significant impact on the leaf growth of lettuce and on photosynthetic activities [22,23]. Legendre and Van Lersel [24] reported the important impact of far-red on plant architecture and its positive influence on plant photosynthesis. Wavelengths shorter than blue fall into the UV range and have been reported to have a positive effect on the accumulation of secondary metabolites in plants. These secondary metabolites often accumulate and perform many functions, including acting as sunscreens in intense radiation environments and chemical protectants against insect herbivory. Ferreyra et al., for example [25], reported that flavonoids were accumulated under the impact of UV-B radiation.

Lettuce is widely cultivated in plant factories under LEDs [26] because of its capacity to adapt to controlled environments. It is often used as a “test crop” in new installations. It has a short growth cycle and a defined rosette shoot shape [27,28]. Several researchers have investigated the impact of light spectra on the growth and development of lettuce [29] and have reported that combined red and blue LEDs exhibit the highest chlorophyll content and photosynthesis rate. However, when monochromatic blue or red LEDs were applied alone, lettuce plants showed growth abnormality and a decrease in the rate of photosynthesis [30]. This finding is in agreement with what was reported by [31], indicating that the use of red LED alone resulted in reduction in biomass, chlorophyll content, carotenoid content and antioxidant levels in lettuce. Others have also reported that red light alone led to abnormality in lettuce, and a combination of 90% red and 10% blue had a positive impact compared to red light only [32]. Furthermore, it has been reported that an increased fraction of blue light at 435 nm in combination with red light at 663 nm at a high irradiance improved the physiological indicators and enhanced the yield of lettuce [33]. It was also reported that the photosynthetic rate and stomatal density depends on the red light/blue light ratio [29]. In this regard, it was reported that increasing R/B ratio from 0.5 to 3 enhanced the level of chlorophyll and flavonoid content, nutrient uptake and water use efficiency of lettuce [5].

The study reported here presents the results of the first experiment to be carried out in a customized PF research installation in Saudi Arabia. The PF was designed by researchers from the University of Plymouth UK and assembled in China and was constructed in a shipping container. The experiment aimed to investigate the impact of a wide range of LED light spectra, including red, blue, green, far red, UV and white on the growth, yield and quality traits of two varieties of lettuce (green and red). Moreover, it investigated whether the relative ratio of blue and red LEDs would impact the growth, physiology and chemical profile of lettuce compared to a full white spectrum. Finally, the impact of supplementing the blue/red growth lights with UV, far-red and green LEDs was also investigated. According to our data, this is the first study to investigate the impact of two types of blues (450 nm and 435 nm) on the growth, physiology and chemistry of two types of lettuces. Therefore, the current paper aimed to provide a deeper understanding of the impact of light spectra on lettuce growth and development.

## 2. Materials and Methods

Seeds of two varieties of lettuce, Carmoli (red lettuce) and Locarno (green lettuce) (Rijk Zwaan, Burgemeester Crezéelaan, De Lier, The Netherlands) were sown and germinated for 14 days in the greenhouse at Muzahimiyah Research Station, King Abdulaziz City for Science and Technology (KACST). Seeds were sown in Rockwool cubes (35 mm) (one seed per cube) and when seedlings produced their first pair of true leaves, they were transferred to the KACST Plant Factory research facility. The KACST Plant Factory facility is a converted insulated modular shipping container where external light has been excluded. Its multi-tier hydroponic growing system consists of gullies for a nutrient film technique (NFT) hydroponic system and is installed with interchangeable LED light units. VitaLink Hydro MAX (HydroGarden, Coventry, UK) nutrients (highly-concentrated A and B formulations) was used as the nutrient solution in the system. The KACST Plant Factory system is divided into several multi-shelf units, each consisting of four tiers 50 cm apart and plants spaced 15 cm apart. The facility was fitted with air conditioning and dehumidification systems and the temperature and humidity were monitored using an Autogrow control system (Autogrow Systems Ltd., Auckland, New Zealand) set at 22 ± 2 °C and 75 ± 5%, respectively. The dark/light period was set at 8/16 h. The facility is fitted with an entry “air shower” and operatives wore clean sterile overalls, shoes and hats to maintain a clean growing environment. No diseases or pests were observed throughout the experiments.

**Figure 1:**
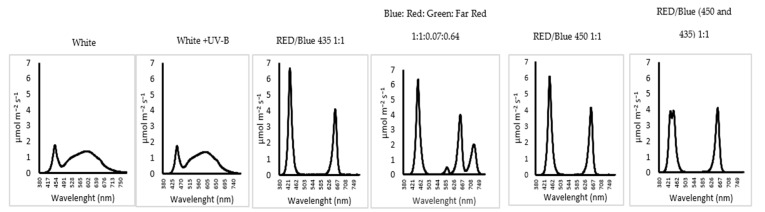
Spectra of the lighting treatments used, as measured by a UPRtek spectrophotometer (Zhunan Township, Miaoli County 35059, Taiwan).

Light intensity (radiant density) was set at 250 ± 10 µmol m^−^² s^−1^ and each LED wavelength was adjustable so that each treatment maintained this light intensity. Six Lumnigrow lighting arrays were used (Table 1) (LuminiGrow, Shenzhen, China) as follows:

1. White (50% cool white + 50% warm light) (W);
2. Red spectral bands with the maximum at660 nm and blue spectral bands with the maximum at 450 nm with ratio (1:1) (R/B450(1:1));
3. Red spectral bands with the maximum at 660 nm and blue spectral bands with the maximum at 435 with ratio (1:1), (R/B435(1:1));
4. Red spectral bands with the maximum at 660 nm and blue spectral bands with the maximum at 450, blue: red (1:1) supplemented by far red and green with ratio (1:1:0.07:0.64) (B/R/G/FR (1:1:0.07:0.64). Green is a wide range wavelength (500–600 nm). Rare red spectral bands with the maximum at 725 nm;
5. White (50% cool white + 50% warm light) + UV-B (0.3 µW cm^−1^) (for an hour/24 h for a week before harvest) (W/UV-B). UV-B intensity was set at 0.3 microwatt.cm^−2^ measured using UV Light Meter UV340B (UV Light Meter, UV340B, Shenzhen Ever Good Electronic Co., Ltd., China). The UV-B was set to switch on during the lighting period of the other wavelengths;
6. Red spectral bands with the maximum at 660 nm and blue at combination of blue spectral bands with the maximum at 435 nm + 450 nm with ratio (1:1), blue: red (1:1). (R/B450-435 (1:1) (Figure 1).

### 2.1. Morphological Measurements

Morphological measurements included plant height (cm); leaf area (LA) (mm^2^) measured using an AM350 Portable Leaf Area Meter (ADC BioScientific, Herts, UK); shoot fresh weight (FW); and dry weight (DW) (g) (after removing the root system) using a sensitive Fisher Scientific SG-402 laboratory balance (Hampton, NH, USA). For dry weight, plants were dried at 70 C° for 96 h [34].

For each stage of development, three replicates (in area) of four plants for each treatment were assessed.

### 2.2. Physiological Measurements

Physiological responses to lighting treatments were measured at three stages of development: 15 days, 30 days and 45 days (final harvest stage) after planting. Physiological measurements included light-saturated instantaneous maximum photosynthetic rate Amax (μmol CO_2_ m^−2^ s^−1^) and stomatal conductance (mol H_2_O m^−2^ s^−1^) measured using an LI-COR 6400 Highly Portable Ambient Photosynthesis System (LI-COR LI 6400XT, Lincoln, NE, United States).

For each stage of development, three replicates of four plants for each treatment were assessed.

### 2.3. Chemical Analysis

Ascorbic acid (vitamin C) was estimated using High Performance Liquid Chromatography (HPLC) (Alliance Waters w2695 Separations Module, Milford, MA, USA). The extraction solution was prepared using 3 g of metaphosphoric acid added to 100 mL of distilled water. A total of 3 g of the plant fresh leaf material was added to the prepared extraction solution, and this was mixed using Glas/Gol vortex (Glas/Gol tools, 711 Hulman St., Terre Haute, IN, USA) at a speed of 70 RPM. The mix was transferred to a water bath (Branaon 8000 West Florissant Avenue, St. Louis, MO, USA) held at 30 °C. The plant extract was then filtered using filter paper (0.45 µm) and the supernatant used for the HPLC analysis using the following column and settings: NewcrcmBH (3.2 × 50 mm, 3 µm), mobile phase: MeCN/H_2_O—50/50%, buffer: H_3_PO_4_—0.5%, flow rate: 0.5 mL min^−1^, UV detection: 275 nm.

Total phenol content was estimated using gas chromatography (GC/MSD Systems—5975C Series GC/MSD System, 5301 Stevens Creek Blvd., Santa Clara, CA, USA). Helium was used as the carrier gas at an average flow rate of 28 cm^3^ min^−1^. A total of 5 g of plant material was ground using a pestle and mortar, 20 mL of methanol was added and the mix was filtered through a 125 mm filter paper. The supernatant obtained was then centrifuged at 1000 rpm for 10 min (Hermle Labortechnik GmbH, Siemensstr. 25, Wehingen, Germany).

HPLC was used for the estimation of total soluble sugar content. The column and settings were shim-pact CLC-NH_2_ (6.0 MM i.d. × 15 cm), mobile phase: Acetonitrile/water (7/3), flow rate: 1.0 mL min^−1^.A total of 5 g of plant fresh material was ground using a pestle ad mortar. A total of 20 mL of distilled water and 20 mL of ethanol were added to the plant material. The mix was filtered through a 125 mm filter. The supernatant obtained was then centrifuged at 1000 rpm for 10 min (Hermle Labortechnik GmbH, Siemensstr. 25, Wehingen, Germany) and used for the soluble sugar analysis.

The total soluble solids of lettuce juice were estimated using a digital portable refractometer (101 model, ATAGO, Japan).

For Chlorophyll estimation, 1 g of plant leaves were ground in 20 mL of 80% acetone using a pestle and mortar. Samples were then centrifuged for 5 min at 1000 rpm min^−1^ in a ROTOFIX 32 (Tuttlingen, Germany). The supernatant (2 mL) was placed in a cuvette and the absorbance was measured at 663.6 (A663.6) and 646.6 (A646.6) using Jenway 7315 (Staffordshire, UK). The formulae are based on the absorbance maxima of each pigment and are dependent on the solvent used. The formulae for samples dissolved in acetone are as follows:

Ca = 12.25 A_663.6_ − 2.55 A_646.6_,

Cb = 20.31 A_646.6_ – 4.91 A_663.6_,

where

A_663.6_ is the solution absorbance at 663.6 nm and A_646.6_ is the absorption at 646.6;

Ca: chlorophyll A, Cb: chlorophyll B, Total C: total chlorophyll.

The values obtained were converted to estimate the chlorophyll content (mg) per gram of fresh weight, following a previously described procedure [30].

Three biological replicates, each consisting of 4 plants (experimental replicates), were applied for each chemical test.

### 2.4. Statistical Analysis

For each lettuce variety, three replicates for each treatment were applied. Each replicate consisted of 4 plants (12 plants in total across the three developmental stages).

Results are presented as means ± standard error (S.E.). All data were subjected to analysis of variance (ANOVA), using Minitab software (version 19) and comparisons of means were made using the least significant difference test (LSD) at 5% level of probability.

## 3. Results

### 3.1. Morphological Parameters

The two varieties of lettuce (green and red) used in this experiment responded differently to the various growing light spectra and are presented separately for ease of interpretation.

**Figure 2:**
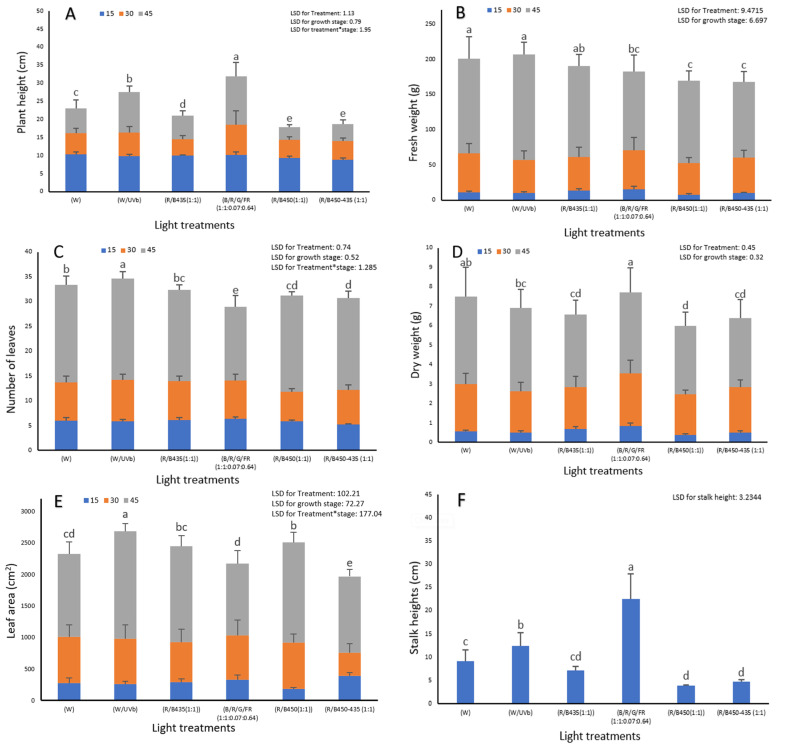
The impact of LED light spectrum on (**A**) plant height (cm); (**B**) plant fresh weight (g); (**C**) number of leaves; (**D**) plant fresh weight (g); (**E**) leaf area (cm^2^) and (**F**) stalk height (cm) of green lettuce (Locarno variety) at three developmental stages, 15, 30 and 45 days from transplanting in the plant factory. Letters indicate significant differences (*p*< 0.05) between treatments within each experiment (* refers to the interaction between treatments).

For green lettuce, all of the various light spectra were capable of producing acceptable marketable lettuce, but some spectra performed better than others. In terms of final yield, white light and white light + UV provided the best crops with most leaves, highest leaf area and greatest fresh weight (Figure 2). R/B450 supplemented by far-red and green (R/B/G/FR 1:1:0.07:0.64) treatments significantly increased plant height (*p* ≤ 0.001) by increasing stalk length. This increase in stalk length indicates a premature flowering response and is an undesirable characteristic for marketable lettuce.

These effects were evident even at early harvests (Figure 2).

For the red lettuce variety, the effects reported above for green lettuce were less accentuated. Whilst R/B450 supplemented by far-red and green (R/B/G/FR 1:1:0.07:0.64) treatments still led to increases in stalk length, the R/B/G/FR 1:1:0.07:0.64 treatment was the only treatment where this was at unacceptable levels for marketable lettuce. The white light treatment provided the most acceptable marketable lettuce.

**Figure 3:**
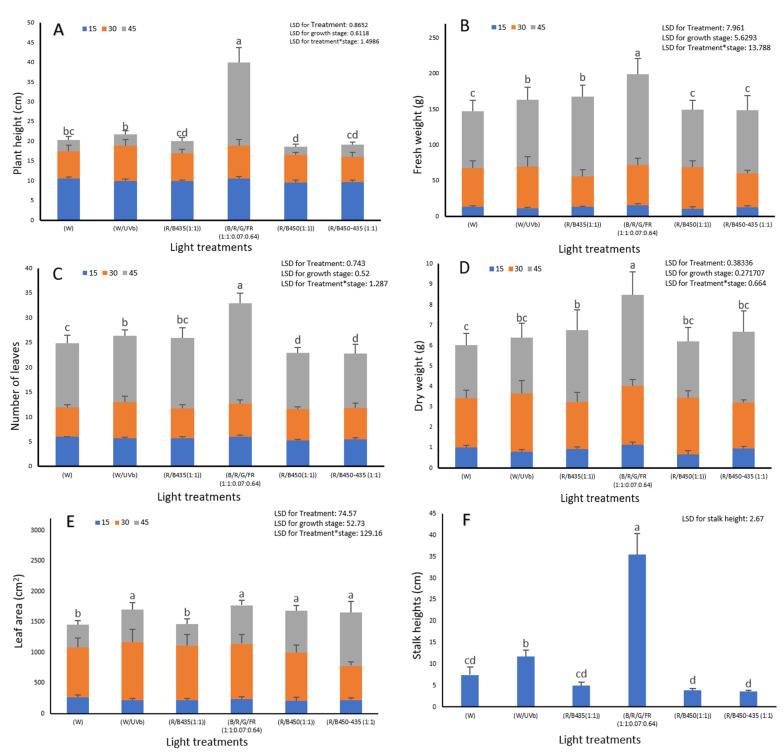
The impact of LED light spectrum on (**A**) plant height (cm); (**B**) plant fresh weight (g); (**C**) number of leaves; (**D**) plant fresh weight (g); (**E**) leaf area (cm^2^) and (**F**) stalk height (cm) of Carmoli variety (red lettuce) at three developmental stages, 15, 30 and 45 days from transplanting in the plant factory. Letters indicate significant differences (*p*<0.05) between treatments in each experiment.

Differences between the various light spectra were much less evident at the early developmental stages and growth rates significantly accelerated in the last 15 days of growth (between 30 and 45 days) (Figure 3).

### 3.2. Physiological Parameters

**Figure 4:**
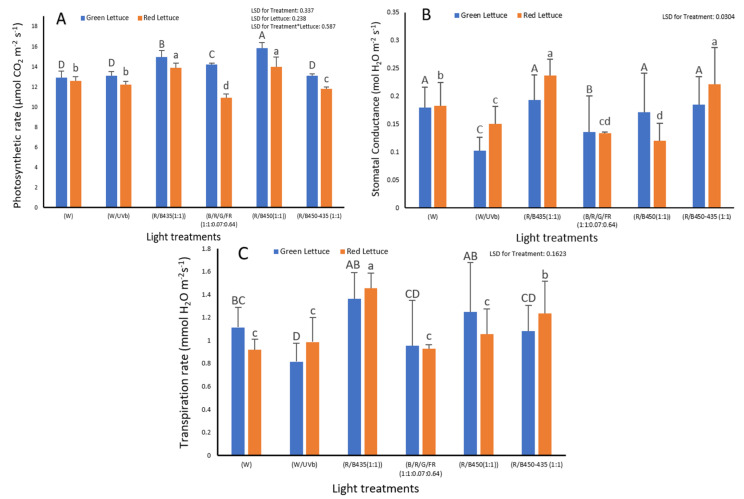
The impact of LED light spectrum on (**A**) photosynthetic rate (μmol CO_2_ m^−2^ s^−1^); (B) stomatal conductance (mol H_2_O m^−2^ s^−1^); (**C**) transpiration rate (mmol H_2_O m^−2^ s^−1^) of green lettuce (Locarno variety) Carmoli variety (red lettuce). Letters indicate significant differences (*p* < 0.05) between treatments in each experiment. (Capital letters are for green lettuce and small letters are for red lettuce.)

Light treatment had a significant impact on the photosynthetic rate of both green lettuce and red lettuce (*p* ≤ 0.001) (Figure 4A). R/B 450 (1/1) and R/B 435 (1:1) resulted in plants with a higher photosynthetic rate in comparison with the use of the white spectrum. Overall, the photosynthetic rate in green lettuce was significantly higher than that of red lettuce (*p* ≤ 0.001).

Light treatment had a significant impact on the stomatal conductance rate (*p* ≤ 0.001). The highest level of stomatal conductance rate was observed when R/B 435 (1:1) light treatment was applied to both green and red lettuce (Figure 4B). There was impact of lettuce variety (*p* = 0.305) and no significant interaction between the lettuce varieties and light treatment (*p* = 0.216) (Figure 4B).

The highest level of transpiration rate was observed under R/B 435 (1:1) treatments (*p* ≤ 0.001). However, there was no significant impact of the lettuce variety (*p* = 0.998) and no significant interaction between lettuce variety and light treatment on transpiration rate (*p* = 0.446) (Figure 4C).

### 3.3. Chemical and Quality Traits

**Figure 5:**
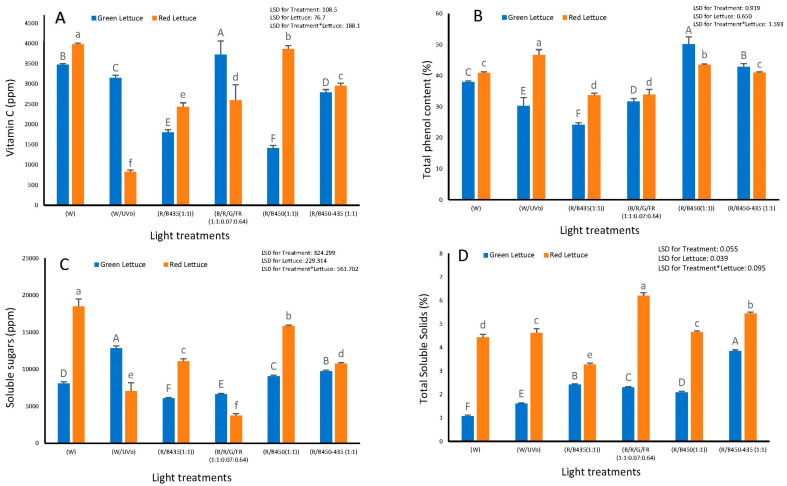
The impact of LED light spectrum on (**A**) vitamin C contents (PPM); (**B**) total phenol content (%); (**C**) soluble sugar content (PPM); (**D**) total soluble solids (%) of green lettuce (Locarno variety) Carmoli variety (red lettuce). Letters indicate significant differences (*p*<0.05) between treatments within each experiment.

A significant effect of light treatment (*p* ≤ 0.001), lettuce variety (*p =* 0.018) and the interaction between light treatment and lettuce variety (*p* ≤ 0.001) on the level of vitamin C was observed (Figure 5A). The highest level of vitamin C in green lettuce was observed under R/B/G/FR (1:1:0.07/0.64), whilst in red lettuce the highest level of vitamin C was observed under R/B 450 (1:1) and white light. Moreover, whilst the addition of UV-B treatment to white light seemed to have a significant negative impact on the content of vitamin C in red lettuce, the lowest level of this vitamin C was observed under R/B 450 (1:1) in green lettuce (Figure 5A).

Total phenol content was also affected by light treatment, lettuce variety and the interaction between light treatment and lettuce variety (*p* ≤ 0.001). The highest phenol content in green lettuce was observed using R/B 450 (1/1) whilst in red lettuce the content of phenol was the highest under white + UV-B treatment (Figure 5B).

There was a significant impact of light treatments, lettuce variety and the interaction between light treatment and lettuce variety (*p* ≤ 0.001) on the level of total soluble sugar (Figure 5C). While white + UV light treatment induced the highest level of soluble sugar in green lettuce, growing red lettuce under the white light had the most positive impact (Figure 5C).

Total soluble solid was impacted by light treatment, lettuce variety and the interaction between light treatment and lettuce variety (*p* ≤ 0.001). R:B450-435(1/1) introduced the highest level to total soluble solids in green lettuce. However, R:B:G:FR (1/1/0.07/0.64) light treatment induced the highest level of total soluble solids in red lettuce (Figure 5D).

**Figure 6:**
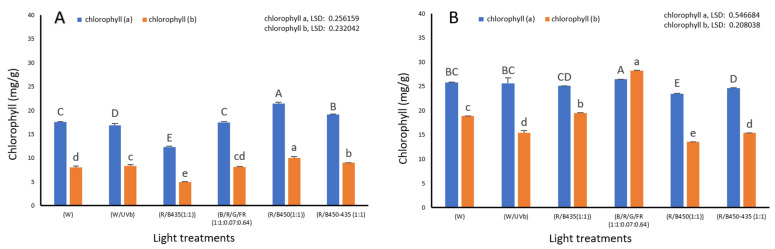
The impact of LED light spectrum on chlorophyll content of (**A**) blue lettuce and (**B**) red lettuce. Letters indicate significant differences (*p*<0.05) between treatments within each experiment (capital letters for chlorophyll (**a**) and small letters for chlorophyll (**b**)).

Light spectrum had a significant impact on chlorophyll content in both green and red lettuce varieties (*p* ≤ 0.001) and overall chlorophyll was lower in green lettuce than in red lettuce. R/B450 (1/1) induced the highest level of chlorophyll A and B in green lettuce (Figure 6A) and R/B/G/FR (1:1:0.07:0.64) provided the highest content in red lettuce (Figure 6B).

## 4. Discussion

To obtain the maximum output from plants grown under CEA, particularly in commercial PF situations, lighting conditions need to be optimized. In particular, the light spectrum can now be manipulated using LED lighting arrays using customised research platforms similar to those installed in the KACST Plant Factory research facility [6,35]. It was shown in the current study that the light spectrum needs can even be variety-specific within a species, indicating that there can be differences in photoreceptors between commercial varieties, a finding supported by other published work [6,7,8,9,10,33].

White light in CEA or PFs is usually supplied using designated white LEDs, which tend to be phosphor-coated blue LEDs generating a full white spectrum including a green rich portion as shown in Figure 1. However, “white light” can also be supplied by employing a combination of monochromatic red, blue and blue LEDs in an array. Both approaches provide a white light and make the facility more user-friendly, but it is not clear which is the best to use for commercial production. Many recent commercial facilities have not opted for white light but instead have invested in red/blue lights to maximize the utilizable radiant energy produced per kW of electrical energy input (the efficacy). The results presented here for two varieties of lettuce clearly demonstrate that both white light and red/blue and red/blue/blue combinations are capable of producing marketable lettuce but significant differences did exist in growth and development rates. White light produced the best plants in terms of commercial yield at the end of the experiment, whereas R/B and R/B/G led to significant stalk lengthening, reducing the marketability. However, the results indicated that under R/B and R/B/G, marketable stage had actually been reached earlier, and although a smaller individual size may have occurred as a result, a marketable product had been achieved some days earlier compared to that under white light. This finding is in agreement with other research that reported the positive impact of white light on the growth and development of plants [36]. The current findings are also in accordance with other research, indicating that lettuce plants grown with spectra that included green light had better growth parameters, such as fresh and dry weights, compared to those grown with light focused in the red and blue region [37]. These findings with lettuce contradict the reports with other species such as basil, which indicate that focusing the lights in the red/blue spectrum promotes growth and yield [7,8], asserting that species can vary in their spectral needs. In our experiment, two R/B LED combinations were tested with the blue either at the 450 nm range corresponding to chlorophyll B absorption or 435 nm corresponding to chlorophyll A absorption as our previous research with basil had indicated a distinct preference for 435 nm over the more conventionally used 450 nm (9). In this case, the wavelength of blue at 435 nm did not promote the growth and yield, as well as the use of blue at 450 nm, which contradicts the findings obtained with basil (9). These species differences appear to be genetically based, which emphasizes that different species respond differently to light spectra, probably due to differences in terms of pigment composition, photoreceptors, plant morphological structure and chemical composition.

Two supplementary non-photosynthetically active LED spectra were also investigated in the current experiment, UV-B and far-red. Whilst the UV-B had no effect on growth and production, the far red did. The far-red supplementation is becoming increasing in interest since Zou (2019) [38], who reported that the total biomass of lettuce was increased by 39% and 25% when the light spectrum was supplemented with FR-Day (through the day) and FR-EOD (end of the day) treatments, respectively. Far red is known to be involved in Phytochrome regulation which in turn regulates morphological and physiological properties and plays a critical role in mediating plant growth and development [39]. In the current study, there was a detectable effect of the far-red supplementation in terms of stem elongation. However, in lettuce production, this is a detrimental quality characteristic that can be associated with bolting, i.e., an early stage towards flowering, which is usually associated with an increased bitter taste and reduced marketability.

Overall, the photosynthetic rate was significantly higher in green lettuce than in red lettuce and light spectrum had significantly different effects on the physiological traits measured. The general trend was that the light spectra which produced the best overall yield (white light) had significantly lower photosynthetic rates and lower transpiration rates when measured. It has to be noted that final yield is an integration of biomass production, partitioning and respiration over time (45 days) whilst photosynthetic rate is an instantaneous measurement at one point in time. It has been reported that sensitivity of a lettuce plant to lighting spectra is determined by its cultivar’s metabolic plasticity [40]. Focusing the lights in the red and blue region (450 nm or 435 nm or a combination of both) had a significant impact on various physiological parameters, including photosynthetic rate, stomatal conductance and transpiration rate, compared with white light. Similar effects were reported in terms of the impact of light spectrum of both basil and lemon balm growth under controlled environment conditions [8,9,10]. However, in the current study, the significant improvements in the physiological traits did not translate into an improvement in the growth rate of either green or red lettuce. It can only be assumed that the morphological structure and metabolic pathways are species specific. More research is needed for a further understanding of the conflicting findings regarding the photosynthesis parameters and growth traits observed, and it is clear that no inferences on final yield can be drawn from instantaneous photosynthetic activity measurements.

Light spectra had a significant impact on various chemical contents of both lettuce varieties. There was also a significant interaction between light treatment and lettuce type in terms of the impact on vitamin C content. While red/blue450 (1:1) treatment significantly increased the content of vitamin C in red lettuce, it has a very negative impact in green lettuce. White light, however, had a positive impact on the vitamin C content of both green and red lettuce. This finding agrees with other reports that suggest that achieving a positive impact on the content of vitamin C is complex, because metabolism of antioxidant properties in lettuce depends on multicomponent exposure of variety, light quality and several other factors related to the growing conditions [41]. Light spectra also impacted the total phenol content in both lettuce varieties, and focusing the lights in the red/blue region significantly increased the accumulation of phenols. The effect of UV-B was of interest as it was predicted that UV would influence secondary metabolism which would include phenolic content. Interestingly, red lettuce responded positively to supplementation with UV, whilst a negative effect of UV was detected in green lettuce. In agreement with the current findings, it has been reported that blue light and ultraviolet-A (UV-A) significantly increase the total phenolic content in soybean [42]. It was also reported that UV irradiation can improve the accumulation of phenolic compounds with antioxidant properties in lettuce cultivated in controlled environment systems [43].

Light treatment had a significant impact on the total soluble sugar in both green and red lettuces. While white light increases the content of these compounds in red lettuce, it was white spectrum supplemented with UV that promoted the highest level of soluble sugar in green lettuce. This is in agreement with previous studies, which indicated that the soluble sugar content of plants grown under RBW treatment is significantly higher compared to those grown under R/B treatments [44]. The higher sugar content could improve the taste and have a positive impact on palatability. Light spectra also had a significant impact on the total soluble content, and this was significantly higher in red lettuce than in green lettuce.

The positive impact of R/B treatments on the chlorophyll content observed in this study agrees with the findings of Naznin (2019) [31], who reported that chlorophyll A, chlorophyll B, and total chlorophyll content of lettuce, spinach, basil, and pepper was significantly increased under the R/B light treatment. However, this is in contrast to previous reports indicating that the total chlorophyll content of lettuce plants treated with blue and red light was less than that of lettuce plants treated with FL (fluorescent light) (white) [45]. In addition, it has been reported that continuous white LED lighting increased chlorophyll content in lettuce [40]. This could be due to genetic variations and also to differences between the experimental conditions in which the other studies were conducted and those of the current research. The positive impact of far red and green supplementation was observed in red lettuce and not in green lettuce. This could be caused by the different compositions of pigment content. It could be also caused by the genetic variations.

UV supplement did not seem to have an overall great impact on the chemical traits of both lettuce varieties. This could be due to the use of low doses for a short period of time (one week before final harvest). Future research needs to include more duration and intensity treatment combinations in order to unfold the possible role of UV in improving the chemical profile of lettuce.

## 5. Conclusions

This investigation set out to compare the effects of various light spectra on the growth and physiological parameters of both red and green varieties of lettuce. Whilst light spectra had a significant impact on various traits, it was apparent that the best spectrum was white light. By using R/B or R/Bl/G spectra, some aspects of physiology could be enhanced, especially instantaneous photosynthetic rate leading to quicker growth, but within the parameters of this experiment, this led to stem elongation and reduced marketability. Attention must be focused on the use of R/B light commercial production systems and recognizing that commercial harvest of a potentially smaller product will be achieved earlier than a conventional white light growing system. This could have commercial advantage if the smaller lettuce size was acceptable to the purchaser. It is worth noting that the addition of far-red wavelengths to the spectrum accentuated the stem elongation in both varieties of lettuce, which needs to be used with caution in commercial set-ups.

There was no clear trend of a light spectrum impact on the general chemical profiles measured despite significant differences being measured. UV-B irradiation gave some indication that it could increase phenolic content, but this effect was only clear in red lettuce. More research is still needed to investigate the mechanism of spectrum impact on the chemical profile of lettuce and on other plant species, since this is clearly species-dependent. Moreover, more studies of possible interactions between light intensity and spectra are recommended.

## Figures and Tables

**Table 1:** The relative ratio of light spectrum in the applied treatments (R refers to red, B refers to blue, W refers to white, G refers to green, FR refers to far-red and UV to ultraviolet.

Treatment	Red (660 nm)	Blue (450 nm)	Blue (435 nm)	Green (520 nm)	Far red	UV-B
R/B450(1:1)	1	1	-	-	-	-
W	0.94	0.53		1	0.13	-
R/B435(1:1)	1	-	1	-	-	-
B/R/G/FR	1	1	-	0.07	0.64	-
W/UV-B	0.94	0.53		1	0.13	+
R/B450-435	1	1			-	-
